# Bone defect treatment: does the type and properties of the spacer affect the induction of Masquelet membrane? Evidence today

**DOI:** 10.1007/s00068-022-02005-x

**Published:** 2022-06-21

**Authors:** Emmanouil Liodakis, Vassilis P. Giannoudis, Stephan Sehmisch, Animesh Jha, Peter V. Giannoudis

**Affiliations:** 1 Trauma Department, Hannover Medical School (MHH), Carl-Neubergstr. 1, 30625 Hannover, Germany; 2 School of Chemical and Process Engineering, University of Leeds, Leeds, UK; 3 Academic Department of Trauma and Orthopaedics, Leeds Teaching Hospitals, University of Leeds, Leeds, UK

**Keywords:** Masquelet technique, Cement spacer, Induced membrane, Bone defect, Open fractures

## Abstract

**Purpose:**

High clinical success rates have been reported with the Masquelet technique in the treatment of traumatic bone loss. An increasing number of studies suggest that various factors can influence the properties of induced membranes. Goal of this systematic review is to answer the following questions: (1) which are the ideal spacer properties (material, surface topography, antibiotic supplementation) to booster the quality and osteogenic potential of induced membranes? (2) what is the ideal time to perform the second-stage operation?

**Methods:**

A systematic search using the keywords “((Masquelet) OR (Induced Periosteum)) AND ((Spacer) OR (Time))” was performed in PubMed, Embase and Cochrane Library according to PRISMA guidelines. Studies published up to the 23rd of February 2022 were included and assessed independently by two reviewers.

**Results:**

Thirteen animal and 1 clinical studies were identified to address the above questions. Spacer materials used were PMMA, silicone, titanium, polypropylene, PVA, PCL and calcium sulfate. With the exception of PVA sponges, all solid materials could induce membranes. Low union rates have been reported with titanium and rough surfaced spacers. Scraping of the inner surface of the IM also increased bony union rates. In terms of the ideal timing to perform the second-stage evidence suggests that membranes older than 8 weeks continue to have regenerative capacities similar to younger ones.

**Conclusion:**

Membranes induced by smooth PMMA spacers loaded with low concentrations of antibiotics showed powerful osteogenic properties. Other materials such as Polypropylene or Calcium sulfate can also be used with good results. Despite current recommendation to perform the second stage operation in 4–8 weeks, membranes older than 8 weeks seem to have similar regenerative capacities to younger ones.

## Introduction

Long-bone defects resulting either from open fractures or from debridement for post-traumatic osteomyelitis represent still a major challenge for orthopaedic trauma surgeons. Various treatment options have been described to treat these defects including autologous bone grafting, vascularized fibular grafts, bone transport, diaphyseal replacement, allografts, titanium cages and the Masquelet technique [1]. Although several animal studies focus on the development of scaffolds/implants to allow guidance of bone formation [2–4], the two clinically most widely used techniques are bone transport described by Ilizarov [5] and the induced membrane (IM) technique described by Masquelet [6]. Bone transport has the advantage of no donor site morbidity, however, the treatment period is prolonged and complicated [7]. On the other hand, the IM technique is technically simple, since implants and approaches are familiar to most orthopaedic trauma surgeons and the union rates are high [8, 9].

**Fig. 1:**
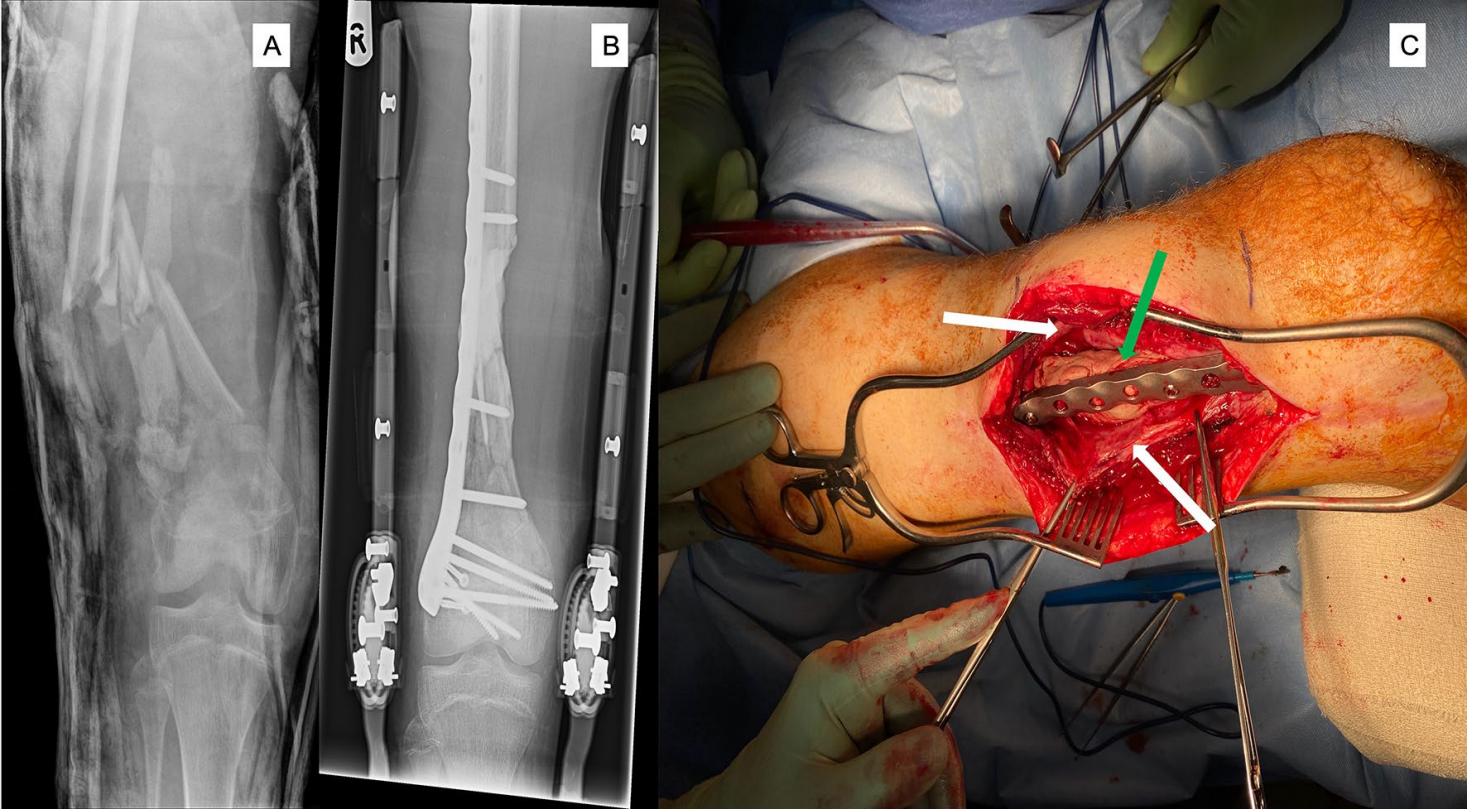
**A** Right distal femoral open fracture in a 18 year-old male patient sustained following a motorbike accident. **B** The fracture following debridement was stabilised with a locking plate and the bone defect was managed with a cement spacer (1st stage Masquelet technique). **C** Intraoperative picture during the second stage and prior to removal of cement spacer (green arrow) demonstrating the induction of the membrane (white arrows)

The IM technique is performed as a 2-stage procedure. In the first stage, the bony defect is stabilized by an internal or external device and a spacer is placed into the defect to manage the dead space and to facilitate the formation of the membrane. In a second stage, 4–8 weeks later, the spacer is removed carefully, and the empty cavity surrounded by the IM (Fig. 1) is filled with bone graft [10]. Induced membranes are not simply a barrier, inhibiting soft tissue invasion into the bony defect, but have powerful osteogenic properties [11–13]. An increasing number of clinical and animal studies suggest that various factors (time to second operation, spacer characteristics, etc.) can influence these properties of induced membranes [13–18].

The goal of this systematic review is to answer the following questions: (1) Which are the ideal spacer properties (material, surface topography, porosity, antibiotic supplementation) to booster the formation and bioactivity of induced membranes? (2) What is the ideal time to perform the second-stage operation?

## Materials and methods

### Search strategy

A systematic search according to PRISMA [19] using the search terms “((Masquelet) OR (Induced Periosteum)) AND ((Spacer) OR (Time))” in PubMed, Embase and Cochrane Library was performed as of the earliest records till 23rd February 2022 by the first and second author. The search in PubMed and Embase was limited to the languages English and German.

### Eligibility criteria

Studies were included if they met the following criteria: (1) studies investigated the Masquelet membrane in relation to the spacer or time; (2) the outcomes included histological and/or immunohistochemical and/or biomechanical and/or radiological data; (3) animal or clinical studies with the full-text paper published before the 23rd  of February 2022.

Studies were excluded if they met the following criteria: (1) reviews, conference abstracts, case reports, letters, or comments; (2) full-text paper written in English or German was not available.

## Results

**Fig. 2:**
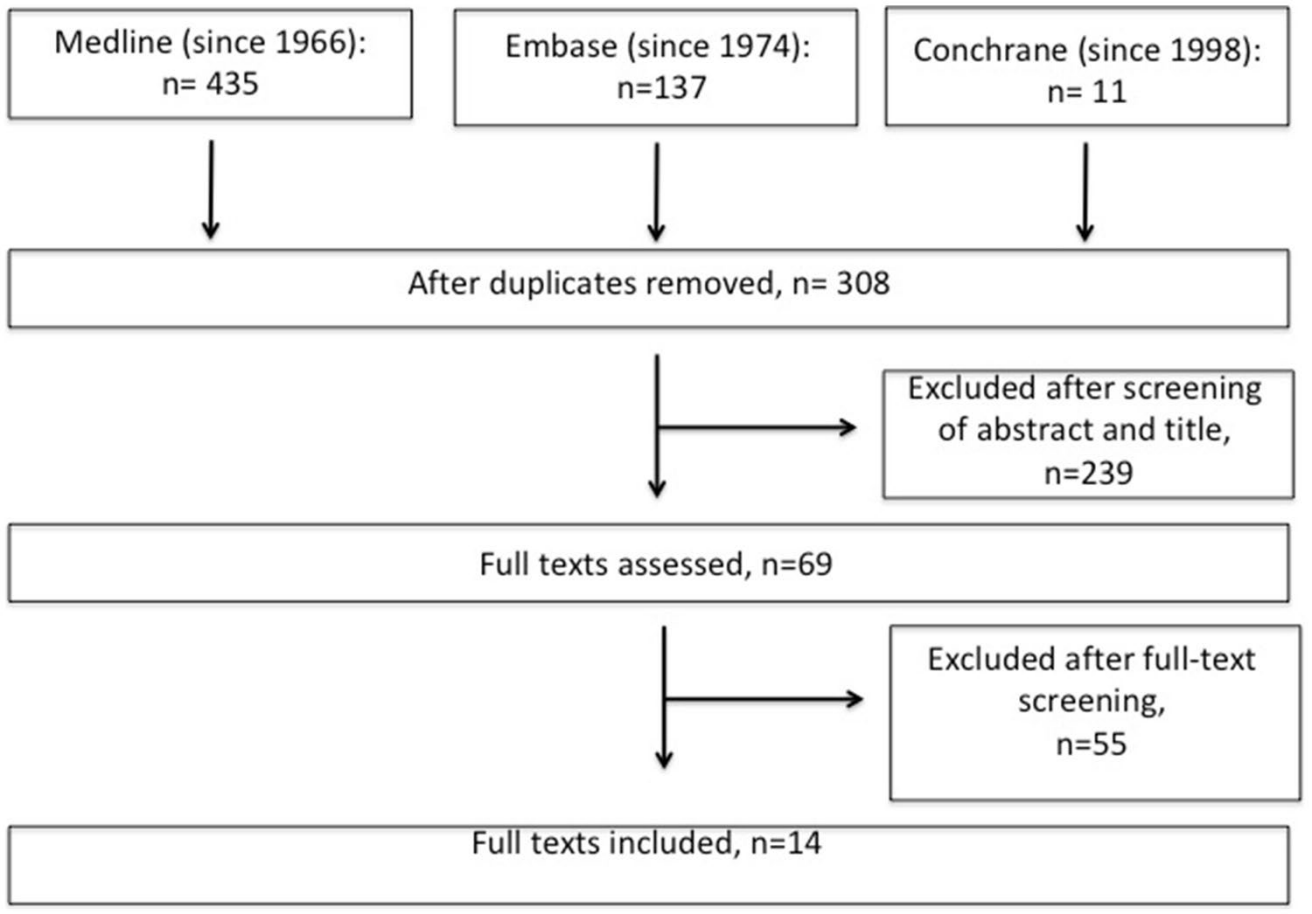
The flowchart of the literature search revealing the outcome for study selection

The flowchart of the literature search is displayed in Fig. 2. Thirteen animal studies [11, 14–18, 20–26] and 1 clinical study [13] have been identified by both reviewers as appropriate to answer our research questions. The most important results of these studies are summarized in Table 1.

**Table 1:** Summary of study details reporting on the type of spacer used and membrane characteristics

Author	Year published	Bone defect- localisation	Bone defect- Size	Fixation device	Spacer implant	Animal types	Sample size	Groups	Time of membrane examination	Membrane characteristics	Conclusion
De Mones [20]	2015	No bony defect	Subcutaneous pockets (15 mm long spacers)	No	PMMA vs Silicone	12-week-old female Wistar *RjHan* rats (average weight 300 g)	28 rats (bilateral)	4 groups (Irradiation of 16 animals with 50 Gray, No irradiation for 12 animals, 2PMMA spacers left, 2silicone spacers right)	9, 13 weeks	Histologic analysis: - PMMA-Induced membranes thicker than silicone. The mean fibrosis score significantly higher for PMMA - The number of vessels per surface area stayed stable in membranes induced by silicone but not for PMMA (reduced vascularisation at 9 weeks after irradiation) - Effect of radiotherapy only for PMMA membranes	(1) Little difference between the membranes induced by the two spacer materials (2) Irradiation seems to affect PMMA more than silicone
Gaio [14]	2018	Femur	6 mm	External fixator	PMMA vs Titanium (Ti)	10-week-old, male *Sprague Dawley* rats	120 rats (right side)	4 groups (PMMA smooth, PMMA rough, Ti smooth, Ti rough)	4 weeks	Tensile testing showed that roughened spacers produced membranes capable of over 40% higher tensile strains at yield No difference between PMMA and Ti	(1) Few differences were seen in the matrix composition, tensile or shrinkage properties of membranes induced by spacers of different materials (2) Altering spacer topography can significantly impact membrane mechanical properties
Mathieu [17]	2021	Femur	6 mm	Internal fixator	PMMA vs Polypropylene	8-week-old, male *Sprague Dawley* rats (average weight 200 g)	50 rats (unilateral)	22 animals used to compare the two materials The rest was used to determine the best time for grafting	2, 4, 6 and 8 weeks	- Histologic analysis: All polypropylene-induced membranes displayed the same two layers observed in PMMA-induced membranes: a cell-rich inner layer and a thick outer layer - BMP-2 was detected in all induced membrane samples from both groups - The 10 week microCT analysis showed no differences between polypropylene and PMMA in bone formation	No microscopic difference between Polypropylene- and PMMA-membranes in histology or cell density
Toth [16]	2019	Femur	6 mm	External fixator	PMMA vs Titanium (Ti)	10-week-old, male *Sprague Dawley rats*	100 rats (unilateral)	4 groups (PMMA smooth, PMMA rough, Ti smooth, Ti rough) 42 animals underwent only the first surgery, 58 two surgeries. Two no membrane negative control groups were included	4 weeks	- Histologic analysis: TI induced membranes were approximately 35% thicker than PMMA-induced membranes, but similar in architecture and expression of BMP2, TGFGb, VEGF, Il6 - The inflammatory factor IL6 was on average 35% higher in the roughened groups than the smooth	(1) Smooth PMMA-induced membranes had better union rates 10 weeks post- implantation (60% vs 9% for rough PMMA) (2) Titanium spacers are likely not a viable option for clinical application in the Masquelet procedure (Union rates 10% for rough Ti vs 22% for smooth Ti) (3) Without the membrane, grafts were resorbed in all cases
Ma [21]	2018	Femur	10 mm	Plate	PMMA vs Calcium Sulfate (CS)	Male *Sprague Dawley* rats, Weight: 260-280 g	60 rats (unilateral)	2 groups	2, 4, 6 and 8 weeks	- VEGF, TGF-β1 and BMP-2 were insignificantly higher in CS-induced membranes than in PMMA - Histologic analysis: Endochondral ossification was observed in CS-induced membrane at the 8th weeks - Western-blot analysis revealed the presence of TGF-β1 and BMP-2 in the induced membranes of PMMA and CS	CS may have the potential to replace PMMA as a novel spacer in Masquelet technique
Sagardoy [22]	2018	Femur	6 mm	Plate	PMMA vs Silicone	12-week-old female *Wistar‐RjHan* rats (average weight 250 g)	32 rats (bilateral)	4 groups: 16 rats received radiation, 16 not. One leg received a PMMA spacer and the other silicone	11 weeks	- Histologic analysis: PMMA and silicone membranes, irradiated or not, had a two‐layer architecture (inner layer: cells, outer layer: vessels and collagen) - Irradiated PMMA membrane was thicker - No influence of radiation on silicone membrane tickness - BMP2 and VEGF expression not modified by spacer implant	- Iradiation decreased their vascular density of the membranes regrdless of the implant - Silicone spacers are able to induce membranes with similar histological characteristics as PMMA
Nau [18]	2015	Femur	10 mm	plate	Different bone cements with or without supplemental antibiotics	8-week-old, male *Sprague Dawley rats* (avarage weight 200 g)	72 rats (unilateral)	4 groups: (1) Palacos + Genta (2) Copal Gentamicn + Vancomycin (3) Copal Gentamicin + Clindamycin (4) Copal space	2, 4 and 6 weeks	- Histologic analysis:Membrane thickness was significantly increased in animals receiving Palacos R + G in comparison to animals which received Copal G + C and Copal Spacem at 6 weeks - Membranes induced by Copal G + C (gentamycin + clindamycin) and Copal Spacem (calci carbonate) were characterized by a significantly increased proportion of elastic fibre in comparison to those membranes induced with Palacos R + G and Copal G + Vafter inductio	- Thickness and proportion of elastic fibres in induced membranes were influenced by the type of cement and the kind of supplemental antibiotics - The positively rated effects of Palacos R + G on the membrane constitution might not be due to a less toxic effect of gentamycin but due to the fact that the gentamicin is only partially released out of the PMMA matrix of bone cements - The antibiotic release from Copal bone cement is more effective. High local concentrations of antibiotics with cytotoxic effects contribute to the impaired membrane maturation
Shah [23]	2017	Femur	8 mm	Custom fixation device was screwed into place	PMMA with or without clindamycin	Skeletally mature male *Sprague–Dawley* rats (275-325 g)	32 rats (unilateral)	4 groups: (1) PMMAA with Clinda + Staph aureus (2) PMMA with Clinda, No Staph aureus (3) PMMA without Clinda + Staph aureus (4) PMMA without Clinda, No Staph aureus	4 weeks	- Histologic analysis showed membranes in the "PMMA without Clinda + Staph aureus" group to be thicker - Expression of BMP-5 in the "PMMA without Clinda + Staph aureus" was significantly downregulated - qPCR analysis of the inflammatory cytokines demonstrates a significant upregulation in the expression of interleukin (IL)-1b, tumor necrosis factor alpha (TNFa), and IL-10 in the "PMMA without Clinda + Staph aureus" group	- Local antibiotic delivery can also be leveraged as an independent means to influence local tissue response for regenerative purpose
Xie [24]	2021	Radius	10 mm	K- wire	PMMA with varying concentrations of vancomycin (0, 1, 2, 4, 6, 8, and 10 g)	New Zealand white rabbits	84 rabbits (unilateral)	7 groups with varying concentrations of vancomycin (0, 1, 2, 4, 6, 8, and 10 g)	2,4 and 6 weeks	- Immunohistochemistry Osteogenic capacity was decreased when the concentration of vancomycin was more than 6 g per cement dose - Real-time PCR Slight increase in the expression of selected genes at low vancomycin concentrations, and relatively lower gene expression when the concentration of vancomycin > 6 g per cement dose	- PMMA spacers loaded with relatively low concentrations of vancomycin (1–4 g per cement dose) did not interfere with the proliferative, osteogenic, and angiogenic capacity of induced membranes, and even promoted their capacity - Spacers loaded with high concentrations of vancomycin (6–10 g per cement dose) had negative effects on osteoblast viability, angiogenesis, and proliferation
Luangphakdy [25]	2016	Tibia	50 mm	Intramedullary rod	PMMA with a smooth vs textured surface	Skeletally mature female goats (5 ± 1 years old)	32 goats (unilateral)	4 groups: (1) PMMA smooth + intact membrane (2) PMMA textured + intact membrane (3) PMMA smooth + scraped membrane (4) PMMA textured + scraped membrane	Week 4 and 12	- Micro-CT analysis showed: (1) Greater bone formation in defects with scraped induced membrane (2) No difference in bone formation between textured and smooth surface - Histologic analysis showed that the most-robust bone regeneration occurred in the scraped induced-membrane group	- Scraping to remove the inner surface of the induced membrane before bone grafting improves bone healing - A textured spacer that increased the induced-membrane surface area had no effect on bone regeneration
Gessmann [13]	2021	Distal Femur/ Proximal Tibia	Not documented	Membrane assessed at the second stage of revision total knee arthroplasty (TKA) or joint arthrodesis due to large femoral bone defects	The spacers were custom-made for each patient using PMMA loaded with antibiotics according to the specific antibiogram. Vancomycin was added in 55 of all the cases, vancomycin/amphotericin was used in 2 cases, gentamicin in 2 cases, and teicoplanin/anidulafungin in 1 case	Clinical study (Human, Age range 35–82)	60 patients (unilateral)	Retrospective study assessing membranes at differing maturation stages (1–16 weeks)	Group 1 = 8–28 days, Group 2 = 29–49 days, group 3 = 50–63 days, and group 4 = 78–113 days	Osteocalcin and osteopontin were found over all time points without significant differences	Membranes older than 8 weeks exert regenerative capacities comparable to the younger ones
McBride [15]	2019	Femur	6 mm	External Fixator	Polymer Polymethyl Methacrylate (PMMA) vs Titanium (Ti) vs Polyvinyl alcohol sponge (PVA)	10-week-old, *male Sprague Dawley* rats	54 rats (27 of those went on to have second stage surgery) unilateral	3 Groups: - (1) PMMA spacer (2) Titanium Spacer (3) PVA spacer	Week 4 and Week 10	Membrane samples were processed for histology to measure membrane morphology, cellularity, and expression of the factors BMP2, TGFβ, VEGF, and IL6. PMMA and titanium spacers created almost identical membranes and phase 1 bone. PVA spacers were uniformly infiltrated with tissue and cells and did not form a distinct membrane. There were no quantitative differences in phase 2 bone formation. However, PMMA-induced membranes supported functional union in 6 of 7 samples while a majority of titanium and PVA groups failed to achieve the same	Spacer material can alter the membrane enough to disrupt phase 2 bone formation. The membrane’s role in bone regeneration is likely more than just as a physical barrier
Henrich [11]	2013	Femur	10 mm	A six-hole 1.5 mm stainless steel mini-plate applied to the lateral aspect of the femur shaft and secured in place with 2 proximal and 2 distal 1.5 mm screws	Polymethyl metacrylate (PMMA) cement	Male Sprague–Dawley rats, 8 weeks old and weighing approximately 300–320 g (*n* = 21; RjHan, Janvier, France)	21 rats assigned into 3 groups (7 each)	1 Group separated into 3 timepoints assessing membranes formed In subcutaneous pockets compared to those formed over the periosteum	Week 2, Week 4, Week 6	Membranes formed around bone defects were similar to those formed in subcutaneous pockets; however, both were significantly different from periosteum with regard to structural characteristics, location of blood vessels and overall thickness Membranes induced at the femur defect (at 2 weeks) and in periosteum contain mesenchymal stem cells (MSCs; STRO-1 +) which were not found in membranes induced subcutaneously. BMP-2, TGFβ and VEGF were significantly elevated in membranes induced around femur defects in comparison to subcutaneously induced membranes, whereas SDF-1 was not detectable in membranes induced at either site. We found that osteogenic and neovascular activity had mostly subsided by 6 weeks in membranes formed at both sites	Osteogenic and neovascular activity in the membranes is maximal between 2 and 4 weeks and subsides after 6. Based on this, better and quicker bone healing might be achieved if the PMMA cement were replaced with a bone graft earlier in the Masquelet technique
Stahl [26]	2021	Femur	6 mm	No	Polycaprolactone (PCL), methyl methacrylate (MMA) eluting PCL (high dose, PCL-MMA and low-dose PCL-MMA) and surgical poly(methyl methacrylate) (PMMA)	10-week-old male Sprague- Dawley (SD) rats	24 rats	4 Groups:- (1) PCL (2) MMA eluting PCL (high dose) (3) MMA eluting PCL (low dose) (4) PMMA	Week 4	A membrane forms around PCL and PCL-MMA, producing osteogenic and angiogenic growth factors and with similar morphology to the IM that forms around PMMA cements. Different concentrations of MMA released from the polymer spacers exerted no statistically significant effect on HUVEC or hMSC proliferation in vitro, nor on the levels of VEGF and BMP2 expressed in vivo; though somewhat increased VEGF levels were observed in PCL-MMA over PCL alone, warranting further investigation. Quantitation of new blood vessels surrounding the implants revealed greater vascularization around the PCL implants in comparison to the high-dose PCL-MMA material, suggesting that the release of unreacted monomer from PMMA surgical spacers is not responsible for promoting the rich vascular network found in induced membrane tissue	The robust vascularized membrane formation around PCL and PCL-based inserts indicates these materials represent interesting candidates for a two-stage IM bone reconstruction study to evaluate their healing potential

Underlined text indicates the tests used to assess IM and bone formation

### Animal models

Rat animal models are the most commonly used in relation to the Masquelet technique. Overall, 10 of 13 animal studies included in this review used rats [11, 14–18, 20–23, 26]. In one study, New Zealand’s white rabbits [24] and in another study, skeletally mature female goats [25] have been used. According to definition of critical size defect (length of defect exceeds the diameter of the affected bone by a factor of 2–2.5) [27], the defect size depended on the size of the animal used. In 9 out of 11 rat models, large femoral defects that ranged from 6 to 10 mm length were created. In one study, no bone defect was created and the spacer was placed in a 15 mm long subcutaneous pocket [20] and in another, a 6 mm long and 1 mm wide slot defect was created in the femur that required no osteosynthesis [26]. In four out of nine rat studies with critical size defects, plates have been used [18, 21, 22], in three studies, an external fixator [14–16], in one study, an internal fixator [17] and in one study, no specification was made [23].

As far as larger animal studies are concerned, rabbits had a 10 mm defect in the radius that was intramedullary stabilized with a K wire [24] and goats had a 50 mm defect at the tibia that was stabilized with an intramedullary nail [25].

### Materials

PMMA has been used in all studies included in this review and acted as the control group in all spacer material comparing studies [14–17, 20–22, 26]. Other materials tested were: Silicone [20, 22], Titanium [14–16], Polypropylene [17], Calcium Sulfate [21], Polycaprolactone (PCL) [26] and Polyvinyl alcohol sponge (PVA) [15]. All solid materials could induce biologically active membranes with similar histological structures to that of PMMA. This was not the case for PVA sponges, that did not induce a membrane [15].

Comparing Titanium spacers with PMMA spacers, PMMA spacers showed better union rates in all studies [15, 16]. According to these results, we conclude that titanium spacers are not a viable option for clinical application.

Polypropylene spacers manufactured from disposable syringes induced bioactive membranes and showed no difference in bone formation at the 10 week microCT analysis [17].

Calcium sulfate spacers did not just induce highly bioactive membranes (VEGF and BMP-2 levels slightly higher than PMMA), but the IM was also thicker than PMMA. Additionally, the calcium sulfate IM showed signs of endochondral ossification, which is unique for this material and makes one-staged procedures conceivable.

Silicone IM showed no significant difference to PMMA as far as thickness of the membrane, vascular density and BMP2 expression are concerned [20, 22]. However, bony union has not been evaluated by these studies. In addition to that De Mones et al. did not create bone defects but placed the spacers in subcutaneous pocket [20]. The results of this study should be looked critically, since it is known that membranes formed in subcutaneous pockets have different bioactivity compared to membranes induced around bone defects [11].

Potential toxic effects of MMA (the major monomeric component of PMMA) have been investigated in a recent study from Stahl et al. [26]. They compared membrane formation around polycaprolactone (PCL) with high-dose MMA eluting PCL, low-dose MMA eluting PCL and PMMA to find robust IM around all groups. No statistically significant effect in regard to levels of VEGF and BMP2 has been detected. From these results, we can conclude (1) that PCL is a material capable of producing a bioactive IM and (2) that the monomer MMA cannot be used to boost the biologic potency of the membrane.

### Spacer topography and porosity

Except for the material itself, each spacer is characterized by its surface roughness, porosity and overall external shape. These properties can have an influence on the effective surface area of the spacer (contact area between spacer and soft tissue) [25].

Roughness of the spacer has been thoroughly investigated by two animal studies [14, 16]. Toth et al. showed that roughening the spacer surface led to increased overall IL6 levels and that higher union rates could be achieved with smooth surfaced spacers 10 weeks post-engraftment in the microCT analysis [16]. Gaio et al. showed that roughening had also an effect on IM biomechanics, creating membranes that are more elastically compliant and failed at significantly higher strains in both directions [14].

Luangphakdy et al. used microCT to compare bone formation between smooth PMMA spacers and textured surface PMMA spacers with 2-mm thick and 2-mm deep linear grooves [25]. Micro-CT analysis found no difference between the smooth and ribbed PMMA spacers as far as total bone volume in the central region of the defect is concerned. Histologic analysis confirmed these results showing that a comparable range of new bone area was measured in both groups. Finally, the authors showed that scraping the inner surface of the IM, increased significantly bone formation.

As far as spacer porosity is concerned, to the best of our knowledge, there are no studies comparing spacers of different porosities to evaluate the quality of IM and bone formation. Shah et al. used high-porosity PMMA spacers [23], reporting induction highly bioactive membranes. The authors used PMMA powder and methylmethacrylate liquid with a carboxymethylcellulose (CMC) hydrogel to impart porosity and PLGA microspheres loaded with or without clindamycin to control drug release [28]. However, the focus of the study was not the porosity itself but the effect of clindamycin on infected bone defects.

### Antibiotics

Three animal studies evaluated the influence of antibiotics and its dosage on the quality of IM [18, 23, 24]. Gentamicin, Clindamycin, and Vancomycin have been analyzed since these antibiotics can be easily mixed with cement powder and reliably eluted from these spacers.

Nau et al. compared commercially available antibiotic supplemented spacers and reported that IM thickness varied significantly in dependency from the antibiotics used [18]. IM formed by a gentamicin (Palacos R + G: 0,25 g gentamicin in 20 g cement powder) and gentamicin-vancomycin spacers (Copal G + V: 0,5 g gentamicin + 2 g vancomycin in 43 g cement powder) were thicker than IM formed around gentamicin-clindamycin supplemented spacers (Copal G + C: 1 g gentamicin + 1 g clindamycin in 42,7 g cement powder) at 6 weeks after surgery. The Copal and Palacos cements used in this are based on the same PMMA formula and were hand mixed in all cases.

Xie et al. explored the effects of different vancomycin concentrations (0, 1, 2, 4, 6, 8, and 10 g per 40 g cement powder) on IM formation in a rabbit radius bone defect model [24]. An obvious decrease in osteogenic and angiogenic capacity measured by immunohistochemical markers (CD31, STRO-1, Ki67) was observed when the concentration of vancomycin was more than 6 g per cement dose. In contrast low concentrations of vancomycin (1–4 g per cement dose) did not interfere with the proliferative capacity of IM, and even promoted their capacity. Even with the highest concentrations, no toxic effects could be observed on the animals.

Shah et al. compared membranes evoked by PMMA spacers with or without clindamycin in contaminated (Staph aureus) and not contaminated bone defects and found the expression of BMP-2 und VEGF to be high in all IM [23]. However, clindamycin treatment of the contaminated defects restored inflammatory cytokine and BMP-5 to the same levels as in the non-contaminated group.

### Time

Plethora of knowledge is available regarding IM changes between the 2nd and 8th week [11, 17, 18, 21]. Animal studies suggest that the thickness of IM is increasing in the first 4 weeks and that the peak of osteogenic and angiogenic activity (measured by Ki67, STRO1 and VEGF) is reached between 2 and 4 weeks. After the 6th week the bioactivity of the IM is subsiding [11].

Little is known about histological and biological changes after this time period. We could identify only one clinical study analyzing IM created after significant longer time periods (up to 113 days) [13]. The authors of this study harvested 60 IM after spacer removal at the second stage of revision total knee arthroplasty. All patients had large femoral defects. The defects were stabilized with intramedullary static spacers. The samples were taken far from the joint structures. ELISA and protein microarray were used to quantify osteogenesis related factors for the different time points. Osteocalcin and osteopontin were found over all time periods without significant differences. VEGF levels seemed to be fluctuating over time but did not show a decreasing tendency. All membranes enhanced proliferation of cultured mesenchymal stem cells (MSC). Thus, the authors concluded that membranes older than 8 weeks retain their biological potency.

### Tests used to evaluate biological potency of the membrane

Although most studies used similar methods to evaluate the quality of the IM, often different outcome parameters were chosen. This makes comparisons and correlations of the results problematic. Here, we summarize the most commonly performed tests in Masquelet-related studies.

Histological analysis was used by almost all studies included in this review article [11, 13, 16–18, 20, 21, 23, 24]. Hematoxylin/Eosin and Elastica van Gieson were the most used stainings (5 μm sections). Next to the histological structure, the thickness of the membrane was measured. IM consisted mostly of two-layers, a thin inner cellular layer and a larger external collagen rich layer [13, 20, 22]. The membrane thickness generally increases in the first 4 weeks, but this can be influenced by various factors (e.g. clindamycin supplemented spacer, radiation) [18, 20]. Mathieu et al. used histologic images to assess cell density, expressed by the number of cell nuclei per mm^2^ [17].

Immunochemistry was also used by most studies to quantify factor expression in the IM. Antibodies specific to growth factors (BMP-2, TGFβ, VEGF) and cytokines (IL6) were commonly used [11, 15–17, 21]. To reveal endothelial cells (mature vessels) immunohistochemical analysis was performed with anti-CD31 antibodies [18, 22, 24]. Immature blood vessels could be identified by von Willebrand Factor antibodies [11, 18]. Cells containing the nuclear protein Ki-67 were also identified with immunohistochemical methods to assess cellular proliferation [11, 18, 24]. Stro-1 antibodies were used to identify MSC [11, 18, 24]. Monocytes that play a major role in the early phases of the induced membrane formation were identified by the expression of the CD14 antigen [18].

The ELISA method has been used by some studies to quantify various bioactive factors such as BMP2 and VEGF [13, 22, 26]. Interestingly, Gessmann et al. co-cultured the IM in contact with growing MSC for 2 weeks before performing ELISA to analyze for supernatants like osteoprotegerin, osteocalcin and bone specific alkaline phosphatase [13]. Western Blot was used by two studies al to quantify the expression of BMP2, TGF-β1 and VEGF in IM extracts [20, 21].

Two studies used cell cultures of Human Bone Marrow Stromal cells (HBMSCs) to assess cell differentiation by measuring alkaline phosphatase (ALP) activity with a colorimetric assay [20, 22]. To achieve that, cell cultures were treated every day with 100 μg of IM lysates. A culture of bone marrow stromal cells in induction medium served as an internal control. Flow cytometry was used by Mathieu et al. to assess the presence of MSCs in IM with various antibodies (Immunophenotyping) [17].

Another two studies used the Real-time PCR to measure the level of expression of m-RNA for inflammatory cytokines (IL-6, IL-10, TNFa) and/or growth factors (VEGF, TGF-β1, BMP-5 and BMP-2) [21, 23].

Micro-CT was used to quantify bone regeneration, bone volume and bone mineral density after Stage 2 of the Masquelet technique [16, 17, 21, 25].

A summary of all the findings related to IM morphological and biological characteristics in relation to the length of time of maintaining the spacer prior to proceeding to the second stage is shown in Table 2.

**Table 2:** Assessment of membrane characteristics/osteoconductive properties in a chronological fashion

Author	Type of study	Week 0	Week 2	Week 4	Week 6	Week 8	Week 9	Week 10	Week 12	Week 13	Other comments
De Mones* [20]	Animal Study						**PMMA-induced non-irradiated membranes** were organised in 2 layers. Superficial layer in contact with the spacer consisted of a layer of fibroblast cells. Deeper plane was composed of collagen, fibroblasts and macrophages. Membrane disorganized and reactive indicating inflammatory infiltration and minor fibrosis. **Silicone induced non-irradiated membranes** were thin, organized in one layer. Fibrous collagen fibres within the induced membrane were organized in parallel and concentric around the spacer			**PMMA-induced non-irradiated membranes** at 13 weeks were organized in two levels **Silicone induced non-irradiated membranes** were organized in two layers, but with a less marked limit observed for PMMA-induced membranes. As for all silicone induced membranes, collagen fibres were orientated in parallel. The mean fibrosis score was significantly reduced in comparison to membranes induced by PMMA at the same time point. Inflammatory infiltration was only seen in a couple of samples, leading to a significantly lower inflammatory score in comparison to non-irradiated membranes by PMMA at the same time point and non-irradiated membranes induced by silicone at the earlier time point Histomorphometric measurements: Thickness of **silicone induced membranes remained stable**, no matter the time point. An influence of the spacer material was seen for non-irradiated membranes, as **PMMA-induced membranes were significantly thicker** than silicone induced membranes at the two time points	**VEGF/ BMP 2** Concentrations of both proteins were too weak to be quantified
Mathieu [17]	Animal Study		Membranes were well structured, showing a clear distinction between the dense inner layer, which included fibroblast-like cells and macrophages, and the outer layer, mainly composed of fibroblasts **Membrane Cell Density** 5144 ± 1093 cells per mm^2^	Similar findings to week 2; however membrane appeared more vascular. **Membrane Cell Density** 4923 ± 1284 cells per mm^2^	Membranes appeared to be more disorganised, connective tissue with many fibrous tissues, including numerous collagen fibres and dense extracellular matrix. **Membrane Cell Density** 4289 ± 493 cells per mm^2^	Membranes appeared to be more disorganised, connective tissue with many fibrous tissues, including numerous collagen fibres and dense extracellular matrix. **Membrane Cell Density** 4099 ± 213 cells per mm^2^		Quantitative and qualitative assessment of bone repair were similar in the two groups. Micro-CT analysis showing no differences in numbers available between polypropylene and PMMA in terms of bone formation Trabecular bone filled the entire defect in most animals, with various callus geometry. A quantitative analysis of the callous volume within the osteotomy region demonstrated no difference with the numbers available in bone volume and bone mineral density between the groups			Induced membranes assessed BMP-2 immunostaining and MSC detection. The immunohistochemical qualitative analysis revealed that BMP-2 staining in induced membranes appeared maximal at 4 weeks, then declined at 6 weeks, and was absent at 8 weeks. No difference noted between groups at any timepoint. The flow cytometric analysis showed that the ex vivo explanted cells from both 2-weekold and 4-week-old membranes displayed the MSC phenotypic profile CD31-, CD45-, CD90 + , and CD73 + . membranes did not generate any MSCs Altogether, these data indicated that the delay between spacer implantation and bone grafting should not exceed 4 weeks. Therefore the second stage should be performed in 4 weeks for two reasons: (1) Although bone turnover marker serum levels were most favourable at 2 weeks, such a short period is ethically questionable and causes a higher risk of further septic complications. (2) 4-week-old induced membranes displayed a high expression of BMP-2
Toth [16]	Animal Study			**Stage 1 Bone Formation Micro-CT**. There were no differences between groups in maximal bone extent length or total bone volumes. However, the proximal total volume was on average 80% larger than the distal total volume **Stage 1 Membrane Thickness & IHC** On average the outer layer was twice as thick as the inner layer. As a whole, Titanium induced membranes were approximately 35% thicker than PMMA-induced membranes. There were no differences in the number of cell nuclei per unit area between groups or layers All examined factors (BMP2, TGFβ, IL6 and VEGF) were more highly expressed in the inner layer than the outer membrane layer. The inflammatory factor IL6 was on average 35% higher in the roughened membrane groups than the smooth		From 6 to 8 weeks there was a noticeable increase in radio-opacity throughout indicating increased mineralization When segregated into their respective groups, the PMMA smooth membranes supported bone regeneration significantly more often than the other three groups. However, there were no statistically significant differences between groups in either micro CT or dynamic histology outcomes		The Micro-CT scan revealed trabecular bone filling the entirety of the defect. Dynamic histology generally mirrored radiography			
Ma [21]	Animal Study	Immediately after surgery both Calcium Sulphate (CS) and PMMA were radiopaque (bone substitute and broken ends clearly visible)	**Morphological structure of CS membrane** Local and mild acute inflammatory reaction and diffuse oedema were noticed, capillaries as well as neutrophils,monocytes, fibroblasts, myofibroblasts, and collagen were seen on the outer surface of the membrane,and synovial-like epitheliums were observed in the inner part of the membrane **Morphological structure of PMMA** Morphological characteristics of induced membranes around PMMA were generally similar to those around CS. The extent of inflammatory and oedema reaction was more severe than in CS group at 2th week	At 4th week, much of the CS appeared fuzzy, dotted and flocculent, indicating partial degradation and absorption. A membrane-like structure appeared around the bone defect while the PMMA material remained relatively clearly visible. **Histological Analysis of Bone Defects**. There were more blue-staining fibrous tissues and micro-vascular sections in both CS and PMMA groups. The lymphocytes and macrophages reduced significantly in number compared to week 2 **Gross Observations and radiographic analysis of the induced membrane in Calcium Sulphate** 4th weeks, the induced membrane was gradually thickening. The outer layer of membrane was still dark red, but a clear border appeared between the membrane and surrounding muscle tissues. The CS partly degraded in the bone defect. Some clear yellow-green liquid was found in the bone defect. The inner layer of the membrane became yellow-green. A small amount of bone callus formed around the broken ends of the bone defect. **Gross Observations and radiographic analysis of the induced membrane PMMA** the induced membrane was generally thickening, and tightly adhesive to the PMMA. Some bone callus formed around the broken ends. The PMMA around the bone defect showed no significant change compared with before. **Morphological structure of Calcium Sulphate membrane** similar to that described in Week 2, and myofibroblasts and collagen fibers mostly arranged parallel to the spacer surface, but less oedematous reaction appeared	**Histological Analysis of Bone Defects**- More fibrous tissues and vascular sections. A few lymphocytes and macrophages apparent. Obvious endochondral ossification was seen at the broken ends. Much more new bone tissue formed near the ends. **Morphological structure of Calcium Sulphate membrane** thickness and fibrosity increased significantly; only a handful of monocytes, neutrophils and small capillaries were observed; larger vessels developed in the outer part of the membrane	**Tracked X-ray Radiographs of Typical Models**. At 8th week, CS was completely degraded and absorbed, membrane-like structure also appeared. The PMMA material showed no significant change compared with previous. **Histological Analysis of Bone Defects** More fibrous tissues and vascular sections were seen. A few lymphocytes and macrophages were also apparent. Obvious endochondral ossification was seen at the broken ends. Much more new bone tissue formed near the ends **Gross Observations and radiographic analysis of the induced CS membrane** Most of the CS degraded in the bone defect. There was an amount of clear yellow-green liquid in the bone defect. A lot of bone callus generated around the plate and the both broken ends, but the bone callus was not yet fully mature, with an obvious boundary with surrounding bone tissue **Gross Observations and radiographic analysis of the PMMA-induced membrane** Membrane around the bone defect partly adhered to the surrounding tissues. Its thickness increased significantly. The PMMA still showed no obvious change compared with before, but some bone callus generated and surrounded the plate and both ends of the bone defect **Morphological structure of CS membrane** Oedema was almost completely absorbed and multinucleated giant cells decreased in number; endochondral ossification, newly formed bone tissue and even mature lamellar bone appeared in the induced membranes **Morphological structure of PMMA membrane** Endochondral ossification or newly formed bone tissue was not observed at the 8th week **Membrane Thickness** As the CS-/PMMA-induced membranes matured, their thickness and fibrocity increased significantly. The CS-induced membranes were generally thicker than the PMMA-induced ones. The thickness of CS-induced membrane (1095 μm) was significantly greater than that of PMMA-induced membrane (842 μm)					The TGF-β1 and BMP-2 protein contents went up gradually at 2, 4 and 6 weeks but declined after 6 weeks. The expressions of VEGF, TGF-β1 and BMP-2 were insignificantly higher in CS groups than in PMMA ones. The expression of IL-6 showed a gradually decreasing tendency in both groups; it was significantly higher in PMMA group than in CS group at week 2 but insignificantly higher at 4, 6 and 8 weeks
Nau [18]	Animal Study		**Membrane Thickness Palacos + Gentamycin** 553 μm **Membrane Thickness Copal + Gentamycin + Clindamycin** 682 μm **Membrane Thickness Copal Spacem** 916 μm		**Membrane Thickness Palacos + Gentamycin** 774 μm **Membrane Thickness Copal + Gentamycin + Clindamycin** 329 μm **Membrane Thickness Copal Spacem** 371 μm						The ratio of immature (vWF +) to more mature (CD31 +) blood vessels increased significantly in groups Palacos + Gentamycin and Copal Gentamycin + Vancomycin whereas no significant alterations were noted in groups Copal + Gentamycin + Clindamycin and Copal Spacem
Luangphakdy [25]	Animal Study			There was greater bone formation in defects with scraped induced membrane compared with intact induced membrane. Mean Bone Volume (tBV) in the central 2.5 cm of the defect was greater in the scraped induced- membrane group versus intact induced membrane When the entire 5 cm defect was considered, the mean tBV in the intact induced- membrane group was 2487.27 mm^3^ and mean tBV in the scraped induced membrane group was 4208.05 mm^3^					The majority of new bone that was present in the sections was composed of cancellous woven bone. Defects containing little or no bone were filled primarily with fibrous connective tissue. There was no evidence of persistence of the original autogenous cancellous bone graft. Histologic analysis showed qualitatively that a comparable range of new bone area measured in the smooth spacer group (range, 9–358 mm^2^) and in the textured spacer group (range, 4–980 mm^2^)		
McBride [15]	Animal Study			All PMMA and Titanium induced membranes had bone extending from the original bone ends to partially cover the spacer surface Comparable volumes originated from the proximal and distal ends that reached similar maximal lengths There were no differences between the two materials. PVA, on the other hand, did not permit the same phase 1 bone growth				Basic mechanical stability was achieved in almost all PMMA animals However, a majority of the TI and Polyvinyl alcohol sponge (PVA) animals did not pass this initial screening indicating that meaningful information could not be gained from mechanical testing Of the quantitative Micro-CT outcomes, there were trends for increased Bone Volume Total Volume, Trabecular Number, and Bone Mineral Density as well as decreased Trabecular Surface in PMMA animals, especially in comparison to PVA animals			All PMMA and TI spacers created similar membranes while PVA had a very different response. At the spacer midpoint between the spacer surface and muscle, all PMMA and TI membranes had two regions that were only distinct under polarized imaging. The inner, layer was significantly thinner than the outer, well aligned layer in both groups In a few samples (*n* = 1–2/PMMA and TI groups) there was a third, disorganized region between the Birefringent (BR) layer and muscle. Collagen type 1 distribution was similar in both regions, but elastin expression was 25–29% higher in the Non Birefringent layer than the BR layer. Structures resembling blood vessels on the Collagen type 1 stained IHC sections were not noted in either the inner or outer layer The BR region of TI membranes was closer in thickness to that of the NB region resulting in an overall significantly thinner membrane. PVA, on the other hand, did not create a similarly structured membrane **Membrane Factor Expression** For all examined factors (BMP2, TGFβ, VEGF, and IL6) there were no significant differences between groups However, all proteins had significantly elevated expression in the NB region. For PMMA-induced membranes the expression of all factors in the NB layer was approximately double that of the BR layer. For TI induced membranes, the differences between the two regions were less (30–85% increase in NB)

Bold text indicates the different types of IM

Bold underlined text shows the membrane characteristics

Index – Please note differing font colour relates to differing properties assessed with respect to each membrane. Papers which presented findings only at one time point were removed from this table. (Morphology/Thickness/Density/Cellularity & Growth Factor release)

^*^Only findings from non-radiated membranes presented

## Discussion

The success of Masquelet technique is based on creating a vascularized soft tissue envelope with similar—but not identical—properties to that of periosteum (“induced periosteum”) [29, 30]. This membrane is not just a barrier that impedes graft resorption but a biologic chamber that secretes various growth factors critical for bone regeneration [31, 32]. To further improve the biologic properties of the membrane, scientific efforts are made to optimize modifiable variables (spacer characteristics, timepoint of second operation) that could influence membrane formation.

### Spacer material

Traditionally, the spacer used during the IM technique is made of polymethylmethacrylate (PMMA), a material well known to most orthopaedic surgeons [33]. PMMA can be moulded in the operating room to suit all defect sizes and morphologies. This gives the operating surgeon more flexibility compared to prefabricated spacers, an important feature, since defect size can change after debridement. In addition, PMMA provides mechanical stability and can be easily supplemented with various antibiotics in various dosages to treat deep infections and prevent biofilm formation [18]. On the other hand, concerns have been raised regarding potential toxicity of PMMA adjuvants and detrimental heat because of the exothermic reaction during cement polymerization [17, 20, 22].

The current literature does not support these concerns since no differences have been detected neither histologically in IM structure nor in cell density between PMMA and other solid materials such as titanium, polypropylene, silicone and calcium sulfate [14, 17, 21]. In addition to that, no significant differences have been recorded in BMP-2 levels and mesenchymal stem cells (MSCs) between PMMA and other solid materials [16, 17, 22]. Stahl et al. also showed that even high concentrations of MMA (the major monomeric component of PMMA) exerted no statistically significant effect on hMSC proliferation in vitro, nor on the levels of VEGF and BMP2 in vivo [26].

Although titanium spacers can induce bioactive membranes the bony union rates are significantly lower compared to that of PMMA, making this material not a viable option currently for clinical use [15, 16].

Polypropylene, on the other hand, evoked bioactive membranes and showed no difference in bone formation at the 10 week microCT analysis [17]. Use of Polypropylene spacers, manufactured from disposable syringes has also been described in a clinical case series study to reconstruct metacarpal bone defects with very good results [34]. Polypropylene, however, cannot be used in clinic as a local antibiotic carrier for treatment of infections.

Calcium sulfate is complete biodegradable and has some osteogenic properties [35]. In addition, it can be used as a vehicle to deliver antibiotics. Ma et al. reported that the calcium sulfate-induced membranes were generally thicker than the PMMA membranes. More interestingly at 8 weeks, some endochondral ossification in calcium sulfate-induced membranes has been observed. No significant differences have been reported as expression levels of VEGF and BMP-2 are concerned. Jiang et al. [36] used calcium sulfate to reconstruct a calcaneal defect in a case report. Eight weeks after introduction of the spacer, a membrane was visible around the spacer and autologous bone graft was used. Although a one-stage strategy was not performed, the authors postulate that for smaller defects a one-stage procedure without use of bone grafts would be possible.

Silicone membranes produce in the animal model membranes with similar characteristics to that of PMMA. However, bone healing has not been evaluated and to the best of our knowledge this material has never been used in clinical practice for a Masquelet procedure.

PVA sponge, on the other hand had a very different response. There was little to no tissue between the spacer material and muscle. Furthermore, the PVA spacer has been infiltrated by fibrous tissues [15].

### Spacer topography and porosity

In a recent animal study, smooth PMMA spacers have been compared with rough PMMA spacers, showing significantly higher union rates for the smooth spacers [16]. Smooth titanium spacers showed also better union rates compared to rough surfaced titanium spacers. Interestingly, roughening has also an effect on IM mechanical properties, creating membranes that are more compliant [14]. IM created by rough surfaces are more likely to be deformed in situations such as graft overfilling or weight bearing, which in turn could affect bone regeneration.

A large animal study on goats compared PMMA spacers with a smooth or textured surface, showing no difference in bone formation [25]. Here, it is important to emphasize that a textured surface is not a rough surface. Scraping of the IM before grafting improved significantly bone healing.

The influence of the porosity of the spacer on IM and bone formation has not been explicitly evaluated via comparative animal or clinical studies. However, we know that a highly porous implants have usually a rough surface and a higher surface area. This could be beneficial for antibiotic eluting spacers since antibiotics and/or other drugs are eluted from the surface and pores of the implant as well as from microcracks within it [37]. Porosity of PMMA can be increased by avoiding vacuum-type mixing devices, since hand mixing introduces air into the cement mixture.

### Supplementation with antibiotics

Bone defects in orthopaedic trauma patients are usually the result of open fractures or deep infections. Therefore, control of infection plays a central role in the treatment of these patients. Ilizarov claimed, “infection burns in the fire of regeneration” and suggested callus distraction to treat bone defects [38]. The IM technique allows surgeons to use the spacer as a tool to introduce antibiotics at the very center of infection. Antibiotics, most commonly supplementing bone cements are clindamycin, gentamicin and vancomycin. In high concentrations, all the above antibiotics can have cytotoxic effects with clindamycin being the most toxic, followed by vancomycin and gentamicin [18, 39–41].

Current literature suggests that local delivery of antibiotics is effective in mitigating deep infections and promotes the expression of growth factors at the IM in relatively low concentrations [23, 24]. However, differences have been reported between commercially available PMMA cements with antibiotic release from Copal bone cement being more extensive than from Palacos [42]. The concomitant use of more than one local antibiotics in combination with Copal cement seems to have a negative effect on the maturation of the IM [18].

### Ideal time point to perform second step procedure

Current clinical recommendation is to perform the second surgery between 4 and 8 weeks post-spacer implantation, assuming the highest IM bioactivity [11, 43]. This narrow time frame has been questioned only by a few studies, that show membranes older than 8 weeks (even years after the 1st stage) to have regenerative capacities similar to younger ones [13, 44, 45]. Highly controlled animal studies are needed to evaluate IM bioactivity beyond the frame of 8 weeks.

## Conclusions

Spacers made of materials offering a rigid barrier with a smooth surface, produce membranes with comparable characteristics in terms of histology, growth factors, and stem cell contents. PMMA is the golden standard for IM technique. Other materials such as Polypropylene or Calcium sulfate can be used.

Supplementing the PMMA spacer with relatively low concentrations of antibiotics (gentamicin, vancomycin, clindamycin) is an effective tool for controlling local infection and can even promote the osteogenic capacity of IM.

Despite current recommendation to perform the second stage procedure in 4–8 weeks, IM older than 8 weeks seems to have regenerative capacities similar to younger ones.
